# Identifying suitable habitat and corridors for Indian Grey Wolf (*Canis lupus pallipes*) in Chotta Nagpur Plateau and Lower Gangetic Planes: A species with differential management needs

**DOI:** 10.1371/journal.pone.0215019

**Published:** 2019-04-10

**Authors:** Lalit Kumar Sharma, Tanoy Mukherjee, Phakir Chandra Saren, Kailash Chandra

**Affiliations:** Zoological Survey of India, Prani Vigyan Bhawan, New Alipore, Kolkata, West Bengal, India; Centre for Cellular and Molecular Biology, INDIA

## Abstract

Different Biogeographic provinces and environmental factors are known to influence the dispersibility of long-ranging carnivores over the landscape. However, lack of empirical data on long-ranging carnivores may lead to erroneous decisions in formulating management plans. The Indian Grey wolf (*Canis lupus pallipes*) is known to be distributed in the vast areas of the Indian subcontinent. However, the actual population estimates are available only for Gujarat, Karnataka, Rajasthan and Bihar. Whereas, its distribution, population and habitat ecology is poorly known from the eastern region. Hence, this article aimed to evaluate the habitat suitability along with landscape connectivity for the species over the two major biogeographic provinces of India, i.e., Lower Gangetic Plains (7b) and Chhota Nagpur Plateau (6b). The present model with significantly higher Area under the curve (AUC) value of 0.981, indicates its accuracy in predicting the suitable habitats and identifying biological corridors by using environmental, topological and anthropogenic variables. Precipitation of the driest quarter and the precipitation of seasonality were the two best performing variables in our model, capable of explaining about 26% and 22.4% variation in the data respectively. Out of the total area i.e. 4,16,665 Km^2^, about 18,237 Km^2^ (4.37%) was found to be highly suitable area and about 3,16,803 Km^2^ (76.03%) areas as least suitable. The corridor analysis indicated that the habitat connectivity was highest in the border line area of the two biotic provinces located in the south-eastern zone via districts of Purba Singhbhum and Paschim Singhbhum of Jharkhand state and Bankura and West Midnapore districts of West Bengal state. Among the Protected Areas (PAs), natural corridors exist connecting the Simlipal National Park (NP)-Satkosia Wildlife Santuray (WLS), Dalma ranges of Chotta Nagpur plateau along with Badrama WLS, Khulasuni WLS and Debrigarh WLS. Differential management through landscape level planning may be helpful in securing the future of the species in the landscape.

## Introduction

India is home to two subspecies of the wolf, i.e. Tibetan Wolf (*Canis lupus chanco*, Gray, 1863), and Indian Grey Wolf (*Canis lupus pallipes* Sykes, 1831) [1,2]. The Tibetan Wolf is distributed in the Himalayan landscape in the elevation range of 3000–4000 m with sub-alpine and alpine conditions. On the contrary, the Indian Grey Wolf is one of the top carnivores in the much of the plans and peninsular region of the country with the varied type of habitats with warm and dry conditions, it occupies grassland, scrublands of semi-arid regions and agro-forestry landscape [3,4,5]. It has been stated that the primary factor behind the establishment of its niche in semi-arid and arid conditions is evolution during the dry period of the Pleistocene [3]. Among the two sub-species, the Indian Grey Wolf is more abundant and presently distributed in isolated grassland ecosystems of Rajasthan in West to West Bengal in East, and from Haryana in North to Karnataka in southern region of the country [5]. Whereas, the Tibetian Wolf is relatively less in number with very confined distribution in the relatively narrow niche in the higher himalayas.

Both the sub-species are losing their range due to a number of threats predominantly increasing incidences of retaliatory killing to reduce human-wildlife conflict [3, 5, 6]. In India, it has been provided highest level of protection by listing the species under the Schedule-I species as per the Indian Wildlife (Protection), Act, (1972). For curbing its illegal trade, the species is listed in Appendix-I of Conservation on International Trade in Endangered Species of Wild Fauna and Flora (CITES), and as per IUCN, the species is classified as least concern considering its wide spread populations of the subspecies globally [7].

In India, studies on Indian grey wolf were largely focused in its western and southern range, but the information on its current distribution and population status in its eastern range have not been evaluated except a short survey from West Bengal [8]. The population of Indian grey wolf have been estimated between 190 and 270 in Gujarat and 253 and 350 in Rajasthan [9]. An estimated 53–85 wolves in 1517 km^2^ area from Solapur, Maharashtra [10]. A relatively recent study from Karnataka estimates a population of 555 wolfs spreading across 123,330 Km^2^ [11]. A population of 2000–3000 wolves is present in the Indian peninsula which seems to be a more realistic population estimate [12].

In the present scenario, most of the large carnivores are experiencing threats and possing managerial challenges due to habitat loss and climate change. As a matter of the fact that the large carnivores require large areas with abundant prey species. But managing such conditions have become a daunting task to the forest managers in the current situation. Both of the sub-species of wolf are known for their involvement in conflicts with humans [6, 13, 14]. In India, conversion of forested land to other land use type and expansion of agriculture into marginalized areas resulting in a reduction of its habitat and prey species [15, 16, 17]. A number of studies are available indicating that the loss of habitat of species is a major factor behind increasing human-carnivore conflicts [14, 18, 19, 20]. The carnivore such as wolf is relatively an opportunistic feeder, and its diet is composed of a variety of species [21]. In agroforestry landscapes, their diet is dominated by domestic species indicating their involvement in livestock depredation. Moreover, in some landscapes due to loss of wild prey or poor abundance prey, the wolves are thriving on domestic species [10, 18]. Hence, in such landscapes, human-wolf conflict is becoming serious threat for its long-term survival.

The populist approach of conservation and management planning through Protected Area (PA) network is not enough for sustaining the viable population of large ranging species in India and elsewhere. Therefore, it warrants the policy planners to develop and adopt a landscape approach in conservation planning, so that consented investments can be made to secure the future of these species and associated ecosystems. For the conservation and management of long-ranging species such as wolf, a better understanding of their distribution and population status is a prerequisite [4, 11]. Effective measures can only be adopted after mapping the species range and habitat assessment. A number of evidences are available where habitat fragmentation and loss of movement corridors resulted in local extirpation of species and loss of genetic vigour among the species populations [15, 16, 17, 19, 22, 23, 24, 25]. Therefore, enhanced knowledge about the biological corridors is imperative for the management planning and sustaining the ecosystems on long-term basis.

Thus, the present study has been conducted to assess the current distribution range and also to map the potential biological corridors of the species in its eastern range of which Chota-Nagpur Plateau (6B) and Lower Gangetic Plans (7B) biotic provinces representing two bio-geographic zones of the country [26].

## Study area

**Fig 1:**
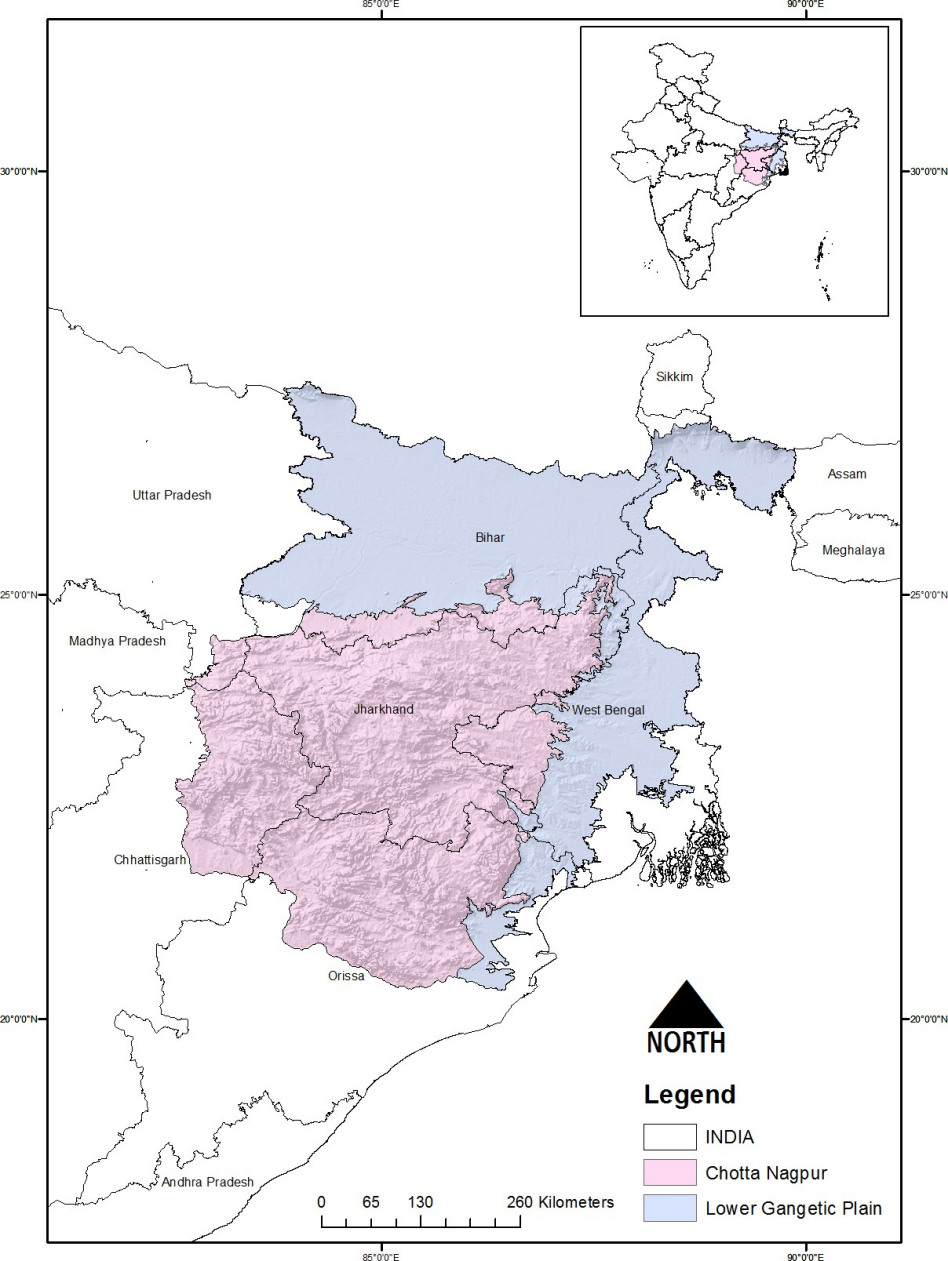
Map showing study area landscape boundary of chotta nagpur plateau and lower gangetic plains provinces in India.

The present study was conducted in two biogeographic provinces namely Chotta Nagpur plateau (CNP) (6b) and Lower Gangetic planes (LGP) (7b) covering much of the Indian Grey Wolf eastern range. These two biotic provinces are part of the Deccan peninsula and Gangetic planes bio-geographic zones respectively, and their classification in based on climatic conduction, soil as well as uniqueness in biodiversity [26] (Fig 1). The LGP cover most of the Bihar, whole of the West Bengal (excluding the Purulia district and the mountain-ous parts of Darjeeling district), eastern region of Orissa and north-eastern portion of Jharkhand States of India. Whereas, the CNP forms the north-eastern edge of the Indian peninsula and the entire plateau can be subdivided into several small plateaux or sub plateaux. It embraces the districts of four states: Bihar, West Bengal, Madhya Pradesh, and Orissa. The study landscape together (CNP and LGP) holds a large network of Protected Areas *viz*., six National Parks and 36 wildlife sanctuaries which in totality account for about 3.47% (14,476.61 Km^2^) of the total area of Chotta Nagpur Plateau and Lower Gangetic Plains [26]. The entire landscape is almost featureless plain except for few mountainous ranges of Malda-West Dinajpur tract, Chotanagpur plateau, and duars of Jalpaiguri (S2 Fig). The mean temperature ranges from 23–38^0^ C with average annual rainfall of 100–150 cm. The vegetation is broadly characterised by dry deciduous forests, tropical and subtropical dry broadleaf forests [27]. The dominated land use type in both the biotic province is agriculture, constituting about 62.10% and 79.23% in CNP and LGP respectively followed by settlements, orchards and water bodies. Increased agricultural and other anthropogenic pressure results in fragmentation and increased disturbance in both the provinces. Recent trends in disturbance profiles also indicating impact of anthropogenic pressure, around 29.11% in CNP and 32.77% in LGP of the total vegetated areas are categorised to be in highest disturbance state [28]. Moreover among the vegetated areas the highly fragmented forest area have increased to about 3.33% and 8.07% in CNP and LGP respectively [28]. The other most prominent large mammalian species present in the study landscape includes *viz*., *Panthera tigris*, *Elephas maximus*, *Tetracerus quadricornis*, *Antilope cervicapra*, *Cuon alpinus* and *Melursus ursinus*.

## Materials and methods

### Ethical statement

Since this study did not involve animal handling and use of biological samples. Therefore, ethical approval was not required. Research permission was taken from Principal Chief Conservator of Forest of West Bengal state of India.

### Study design and data collection

**Fig 2:**
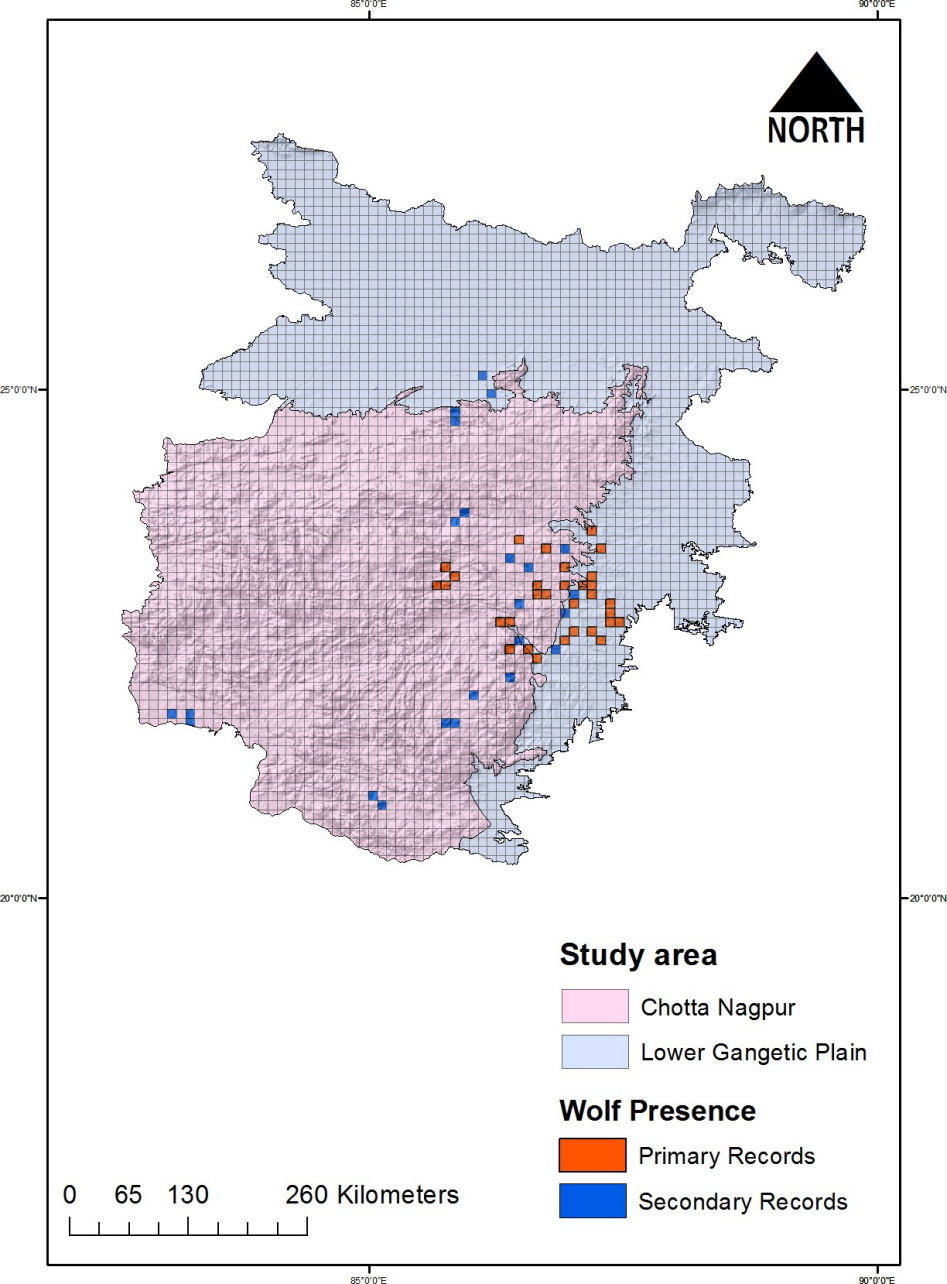
Map showing sampling grids in the study landscape. The red colour grids are those where presence was recorded in primary data, and blue grids presence was based on secondary records.

We have used both primary as well as secondary source data for mapping the habitat suitability as well as identifying biological corridors for the species. The primary data of species observations (physical locations) was collected during 2015–2016 under the programme on status assessment of Indian Grey Wolf in West Bengal, Jharkhand and adjoining areas of Zoological Survey of India, Kolkata (ZSI). Whereas, the secondary data was extracted from the historic records of ZSI and Global Biodiversity Information Facility (GBIF) database (www.gbif.org). A combination of primary as well as secondary data was used to generate species presence information throughout the range of grey wolf in CNP and LGP. For collecting the primary data, field surveys were conducted after dividing the study landscape into 10 X 10 km grids, a line transect of 2–5 km was travelled in selected grids with grey wolf habitats (Fig 2). A total of n = 31 primary wolf location grids along with n = 21 secondary wolf location were visited for recording direct as well as indirect observations (scat, pug marks, denning sites, livestock depredation). A total of n = 126 presence records were gathered during the study period from primary as well as secondary data. For all species presence location information such as GPS location, habitat type, distance to water, distance to the road was recorded [9, 10]. Opportunistic night survey**s** were also conducted from 1800hrs to 2200hrs in areas where the local communities reported wolves.

Additionally, the opinions of experienced field staff (n = 11) served for more than two decades in forest and wildlife department was also gathered concerning with presence and absence of the species (S6 Fig).

### Identifying suitable habitats in CNP and LGP

For identifying suitable habitats for wolfs in the study landscape, we have attempted the ensemble approach implemented in VisTrails pipeline of SAHM package [29]. In the ensemble approach we have combining the five different models namely, Boosted Regression Tree (BRT), Generalized Linear Model (GLM), Random Forest (RF), Multivariate Adaptive Regression Splines (MARS) and Max-entropy for computing the ensemble probabilities. But the results of different modelling algorithms were of similar nature and also all models resulted with AUC < 0.9. Further, for the ensemble approach all model where selected where cut off value for enabling was 0.9. But we suspected an over prediction of the suitability, considering the historical distribution and habitat ecology of the species. Moreover, the over predictions of the ensemble model were influencing our circuit model and making it more ecologically irrelevant, since we were dependent on the suitability output for creating the conductance surface. Therefore, considering the issue related to the over prediction and for making the output more ecologically relevant we drooped the ensemble approach and used the Maxent model in the present study. Ultimately for sake of giving the most accurate and realistic result for the landscape level planning we adopted the maximum entropy based modelling using the software MaxEnt version 3.3.3k [30, 31]. The MaxEnt program provides the probability of occurrence of a given species, ranked from 0 (least likely occurrence) to 1 (most likely occurrence) [32]. The modelling has been executed following subsampling technique with 100 imitations and Receiver Operating Characteristics curve (ROC). The Area under the curve (AUC), has been calculated using 10,000 random background points as pseudo-absences [33]. As a matter of fact selection of the key environmental variables is decisive in determining the habitat niche of a species [34, 35, 36]. Hence, we selected those variables which are fund to be important for the study species [37]. We started with a total of 19 bioclimatic variables (BIO1 to BIO19) along with topographic (elevation, slope and aspect) and linear features (distance to the road, distance of river) and classified forest cover maps (S3 Table). The bioclimatic variables were downloaded from the worldclim database (http://www.worldclim.org/bioclim), ArcGIS 10.6 software (ESRI 2018) was used for calculating the Euclidian distance from river and road and for generating topographic variables (slope, aspect and elevation) using Advanced Spaceborne Thermal Emission & Reflection Radiometer (ASTER). The Landsat 8 data was used for generating the forest cover raster which was further classified into four forest density covers viz., dense forest, moderate dense forest, open forest, scrubs and non-forest types [38]. The Maximum Likelihood Algorithm was used to detect the forest cover classes and the accuracy assessment of the classified image with error matrix was both generated in ArcGIS 10.6. Overall accuracy, user and producer accuracy along with the kappa coefficient were then derived from the error matrices. All the variables were re-sampled at 1km resolution and were converted to ascii (raster) format using ArcGIS 10.6 (ESRI®, CA, USA) Spatial Analyst Extension [39]. The spatial multi-collinearity among the variable was tested using the ENM tool Version 1.3 and the variables with Pearson Correlation Coefficients (r) more than 0.8 were dropped from the analysis [40]. Finally, 12 spatially independent predictors were used for identifying the suitable habitats of wolf in the landscape (Table 1). The model accuracy was assessed by using the Jackknife test in which all the variables were considered independently to measure their relative and absolute contribution to the model. Further, for evaluation of the model, 70% of the species presence sites were used as training data and the remaining 30% was for testing the statistical significance [41]. The threshold value based on the AUC of the ROC ranges from 0 to 1, the AUC score of 1 indicates perfect prediction, with zero omission. However, the values equal to 0.5 indicates random prediction, while AUC values 0.8< AUC<1 were treated as good; 0.7<AUC<0.8 as fair and AUC less than 0.7 poor prediction [42]. The resulting habitat Suitability classes were categorized into four classes’ viz., least suitable, low suitable, moderately suitable and high suitability having the omission ranges from, 0 to 0.060, 0.061 to 0.20, 0.21 to 0.40 and 0.41 to above, respectively for the species in the study landscape.

**Table 1:** List of 12 selected variables out of 23 variables after multi-collinearity analysis for habitat suitability modelling for Indian Grey Wolf (*Canis lupus pallipes)* in the study landscape (Cotta Nagpur Plateau and Lower Gangetic Plain).

Variables	Code	Type
Bio 1 = Annual Mean Temperature	Bio_1	Continuous
Bio 6 = Minimum Temperature of Coldest Month	Bio_6	Continuous
Bio 10 = Mean Temperature of Warmest Quarter	Bio_10	Continuous
Bio 11 = Mean Temperature of Coldest Quarter	Bio_11	Continuous
Bio 13 = Precipitation of Wettest Month	Bio_13	Continuous
Bio 14 = Precipitation of Driest Month	Bio_14	Continuous
Bio 15 = Precipitation of Seasonality (Coefficient of Variation)	Bio_15	Continuous
Bio 17 = Precipitation of Driest Quarter	Bio_17	Continuous
DEM = Digital elevation data (m) from Advanced Spaceborne Thermal Emission & Reflection Radiometer (ASTER)	Bio_DEM	Continuous
River = Euclidian distance (m) from River	Bio_River	Continuous
Road = Euclidian distance (m) from River	Bio_Road	Continuous
Forest Cover 1. Dense forest 2. Moderate dense forest 3. Open forest 4. Scrubs 5. Non-forest 6. Water	Bio_veg	Categorical

The relation between mean suitability, range suitability and sum of suitability score were obtained for all PAs of the area. These values were derived by calculating the zonal statistics for all 42 protected areas extracted from the model output.

### Landscape connectivity and Corridors in CNP and LGP

The Circuitscape software (version 4.0) was used for understanding the connectivity among the habitats of the wolf in the study landscape, which is based on the circuit theory and has been applied in a number of studies aimed at mapping intuitive ecological connections between the habitat patches [43, 44, 45, 46, 47, 48]. We established the connections among the habitat by assessing the conductance values of the raster surface. The higher value of conductance indicates greater movements among the suitable habitats [44]. We used the suitability score of the landscape to develop connectivity model for the species [37, 49]. The most accepted methods for connectivity modelling, are based on graph theory, comprises habitat patches and habitat links connecting the patches [50], followed by another approach i.e. Least Cost Method (LCM), which helps to identify the least resistance path between two points across a cost surface, but LCM have limitations path and actual distance travelled by the species [51, 52]. In contrast to the LCM method circuitscape does not assume that animal drive according to preceding information of the surroundings, but is based on random walks [53]. Therefore, we utilized circuit theory approach as it predicts multiple paths of current flow between different habitat nodes [50, 54]. The Circuitscape requires focal nodes which represents the points between which the connectivity is going to be modelled along with the habitat map reflecting the permeability of each cell, which usually referred to as resistance or conductance value. This conductance value is required for current flow. In the present study, we have evaluated the pair wise electrical resistance value by running the current flow between individual pairs of nodes [53]. We use habitat suitability model output as conductance layer and 22 nodes to run the pairwise connectivity model [55, 56, 57]. Selected nodes were having confirmed wolf presence from the survey data and are well spread throughout different habitat types found in the biotic provinces. We have not used all nodes for running the connectivity model so that complexity can be minimised. The resulting current density map shows cumulative loaded of current flowing through the nodes as a whole, which further characterizes the critical connective areas between nodes. The higher concentrations of current between nodes reveals routes by which animals more likely to move. Locations, where current flow is high or there is no alternate route for current flow is depicted in the model, acts as pinch points or a bottleneck to movement of the species. Such areas are of high conservation priorities and loss of which may have profoundly impact the landscape connectivity for the species.

## Results

A total of n = 126 spatially independent presence locations of the Indian Grey Wolf were recorded during two field surveys carried out in the year 2015–16. Out of which n = 32 presence locations were collected from the questionnaire surveys and secondary sources, i.e., old records of Zoological Survey of India, Kolkata, interview forest staff and GBIF database. A total of 360 ground truth points were used for accuracy assessment equally divided in to forest cover classes. The overall accuracy and the Kappa coefficient of the classified forest cover image was found to be 88.61% and 86.30% respectively where SE of kappa was 0.020 (S1 Table) (S1 Fig).

**Fig 3:**
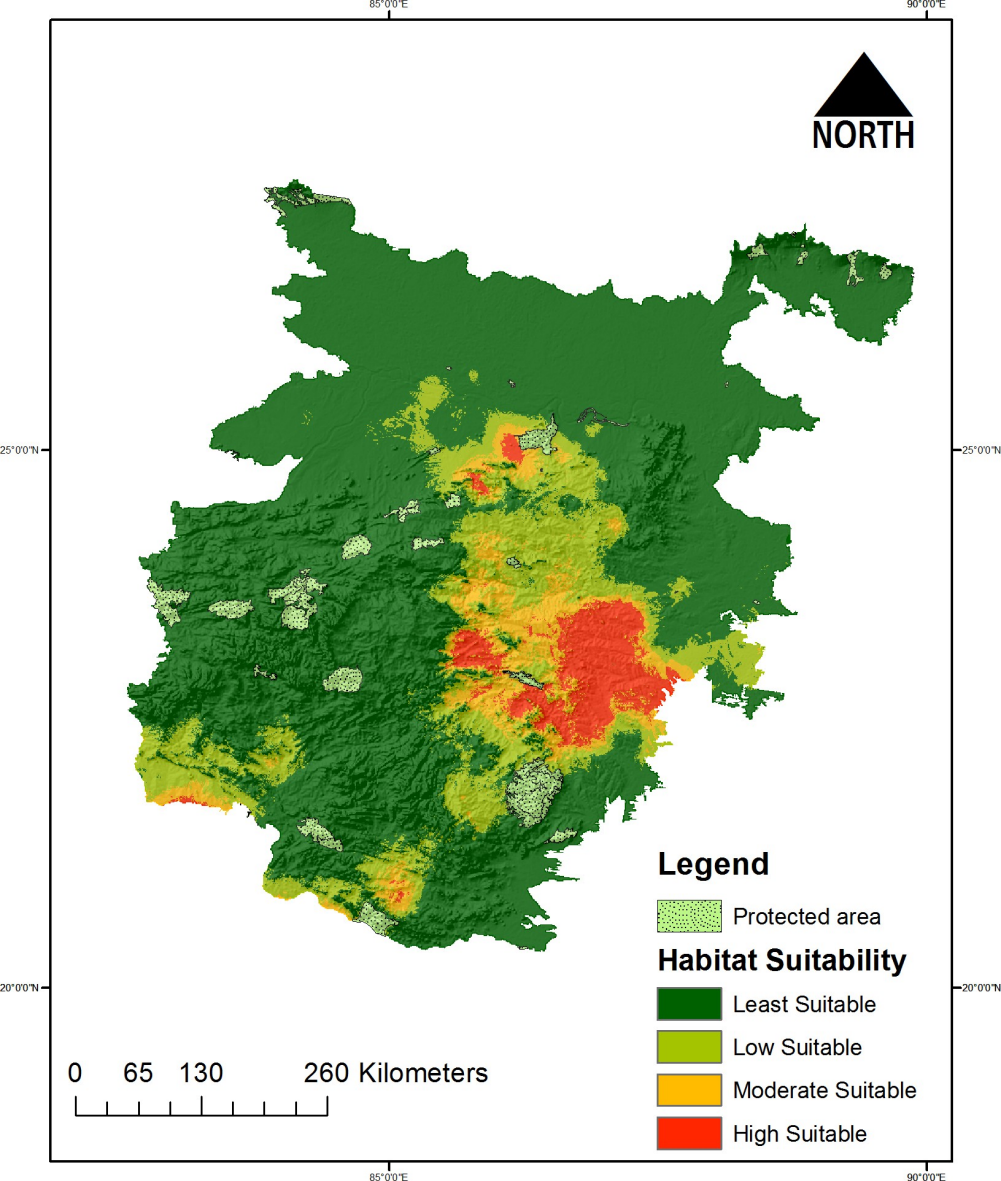
Probability distribution map showing the suitable habitats for *Canis lupus pallipes* in the study landscape.

The model predicted suitable habitats of the wolf in Bankura, Purulia, Midnapore districts of West Bengal, Janjgir Champa, Raigarh, Singhbhum districts of Jharkhand and Sonepur and Angul districts of Orissa states. Further, the present model predicted that out of 42 PAs in the study landscape only 5 PAs such as Dalma WLS, Debrigarh WLS, Bhimbandh WLS, Satkosia and Simlipal Tiger reserve possess suitable habitat of the species in the landscape. However, much of the species suitable habitat exists outside the PA network (Fig 3).

The receiving operating characteristic curve (ROC) value of the present model was 0.981 with a standard deviation of 0.007 (S3 Fig), indicating the importance of selected variables in predicting the suitable habitat of *Canis lupus pallipes* in the study landscape. Among all predictors, the precipitation driest quarter and precipitation of seasonality (Coefficient of Variation) were the two best performing variables and were capable of explaining about 26% and 22.4% variation in the data respectively (S4 Fig). The linear predictors such as distance to road and distance to river/stream were found to be less useful and accounting only for 1% and 1.1% of explained variations in the model (S4 Fig). The response curves of Annual Mean Temperature, Mean Temperature of Coldest Quarter and Minimum Temperature of Coldest Month shows a positive relationship with the logistic output probabilities. However, Precipitation of Wettest Month, Precipitation of Seasonality (Coefficient of Variation), Precipitation of Driest Quarter and elevation were negatively correlated with the logistic probability (S5 Fig).

**Fig 4:**
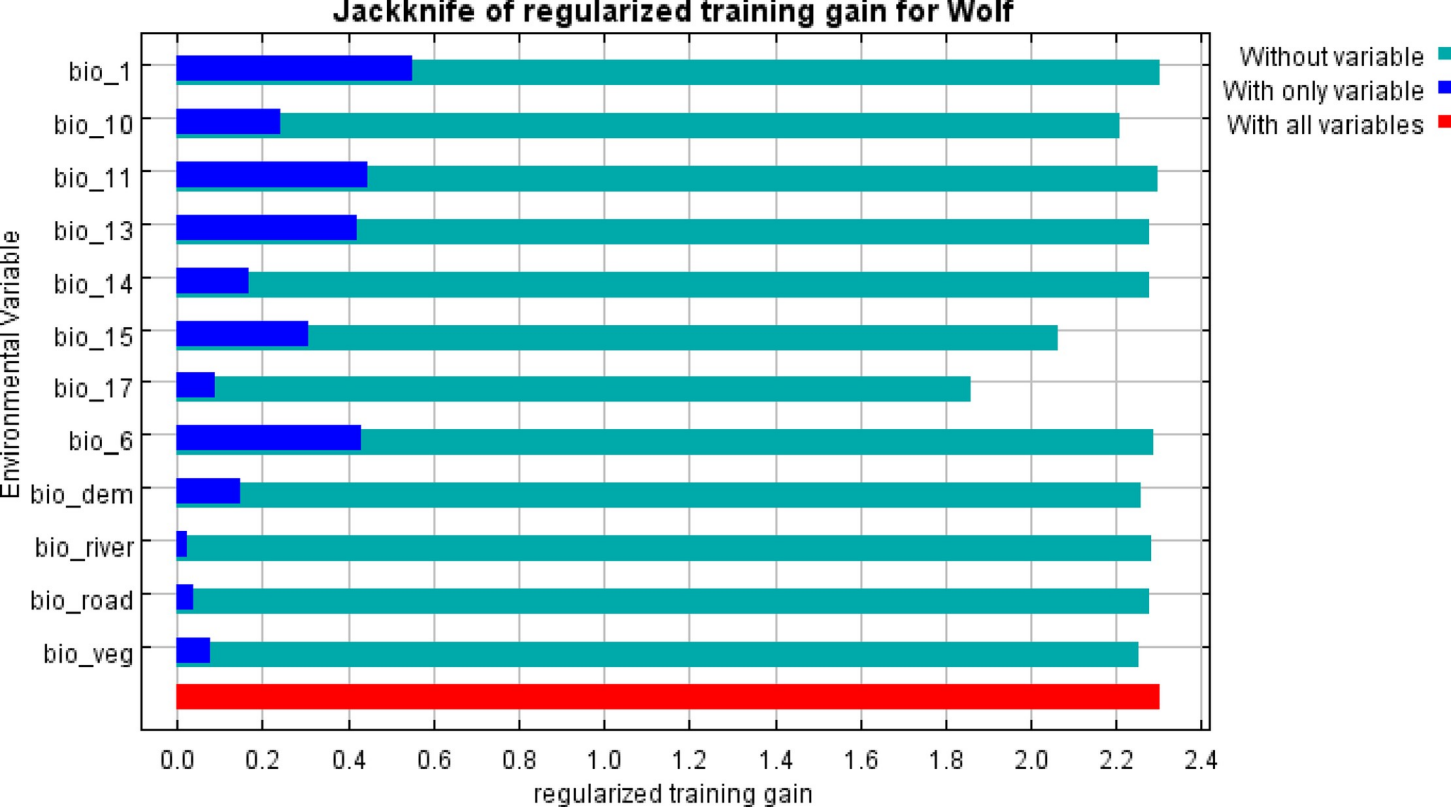
Jackknife test for all the twelve variables. Blue bar = Shows importance of each variables in explaining the variation in the data when used separately. Green bar = loss in total model gain when the particular variable was dropped, signifies the presence of unique information necessary for explaining the model. Red bar = total model gain.

The Jackknife test indicates that the regularized training gain for study species in the present model showed the highest gain when the annual mean temperature was used in isolation for running the model. Whereas, the training sample gain was lowest after omitting the Precipitation of Driest Quarter from the model, indicating its imperativeness in identifying the suitable habitat of the study species (Fig 4).

**Fig 5:**
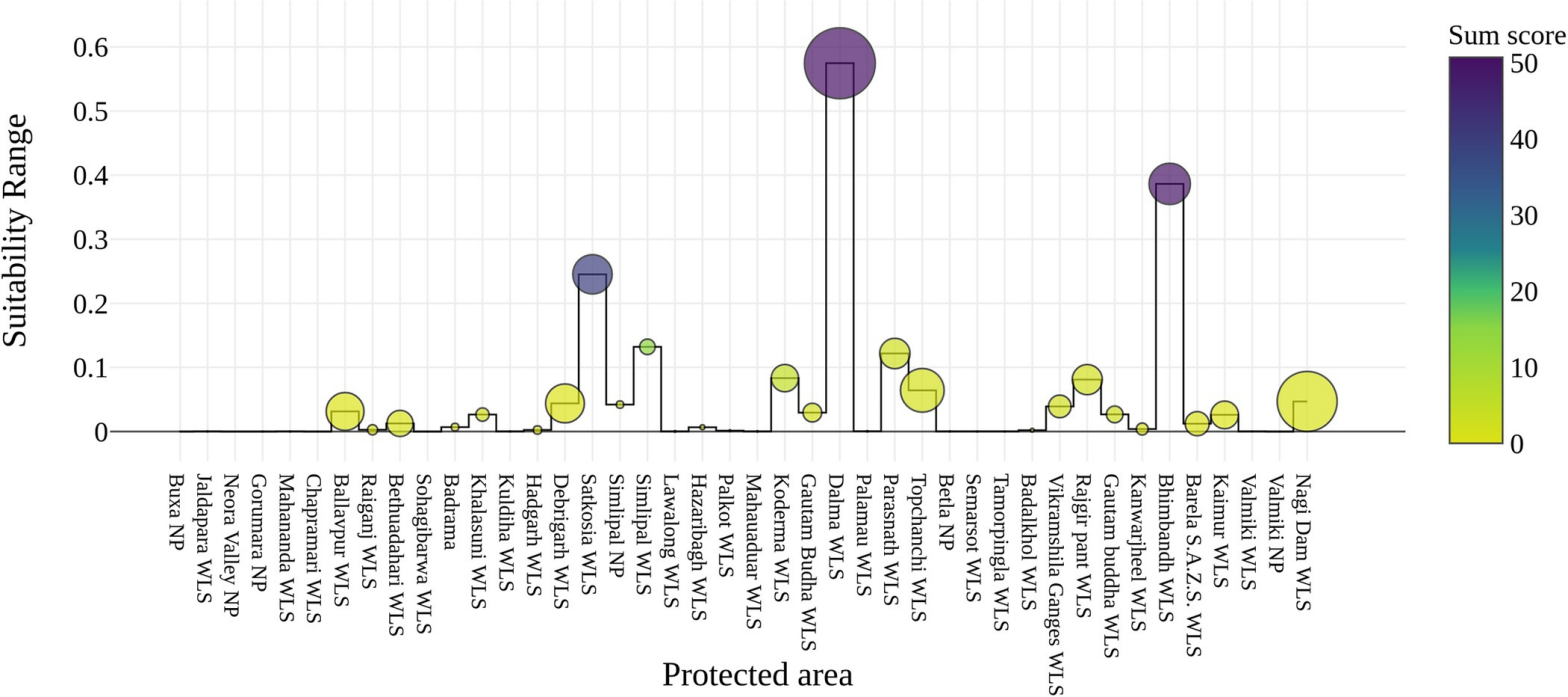
Relation between mean suitability, range suitability and sum of suitability score obtained by PAs. Values were derived from the zonal statistics calculation for all 42 protected areas extracted from the model output. X axis = Protected area, Y axis = Suitability range score for each PAs. Color ramp depicts the sum value of suitability score size of circle represents mean suitability values obtained by respective PAs.

Out of the total area of the study landscape (4,16,665 Km^2^), about 18,237 Km^2^ (4.37%) is classified as highly suitable area, followed by 22,801 km^2^ (5.47%) under moderate suitable 5,88,24 km^2^ (14.11%) in low suitable and about 3,16,803 km^2^ (76.03%) areas as least suitable for wolf (Fig 3). The model also identified that out of 42 protected areas under the CNP and LGP landscape, Satkoshia Tiger Reserve, Simlipal NP, Dalma WLS, Bhiambandh WLS, Nagi Dam WLS and Koderma WLS are the few which possess suitable habitat for the species. Considering the mean value of the suitability score Dalma WLS score the highest of about 0.166 followed by Nagi Dam WLS with 0.119 and Satkosia, Bhimbandh and Debrigarh scoring about ~00.5. Interestingly while summing all the suitability scores among PAs; was highest for Dalma WLS (50.07), followed by Bhimbandh WLS (50), Satkosia WLS and Simlipal NP resulted with a value of 39.75 and 14.17 respectively. The highest suitability score produced by the present model is 0.91 but when considering the max suitability scores under the PAs highest score was only about 0.575 in Dalma WLS followed by Bhimbandh WLS, Satkosia WLS and Simlipal NP scored maximum suitability score 0.398, 0.245 and 0.132 respectively. This indicates that most of the very high suitable areas in the study landscape falls under the non-protected and territorial ranges (Fig 5) (S2 Table).

### Potential corridors in CNP and LGP

**Fig 6:**
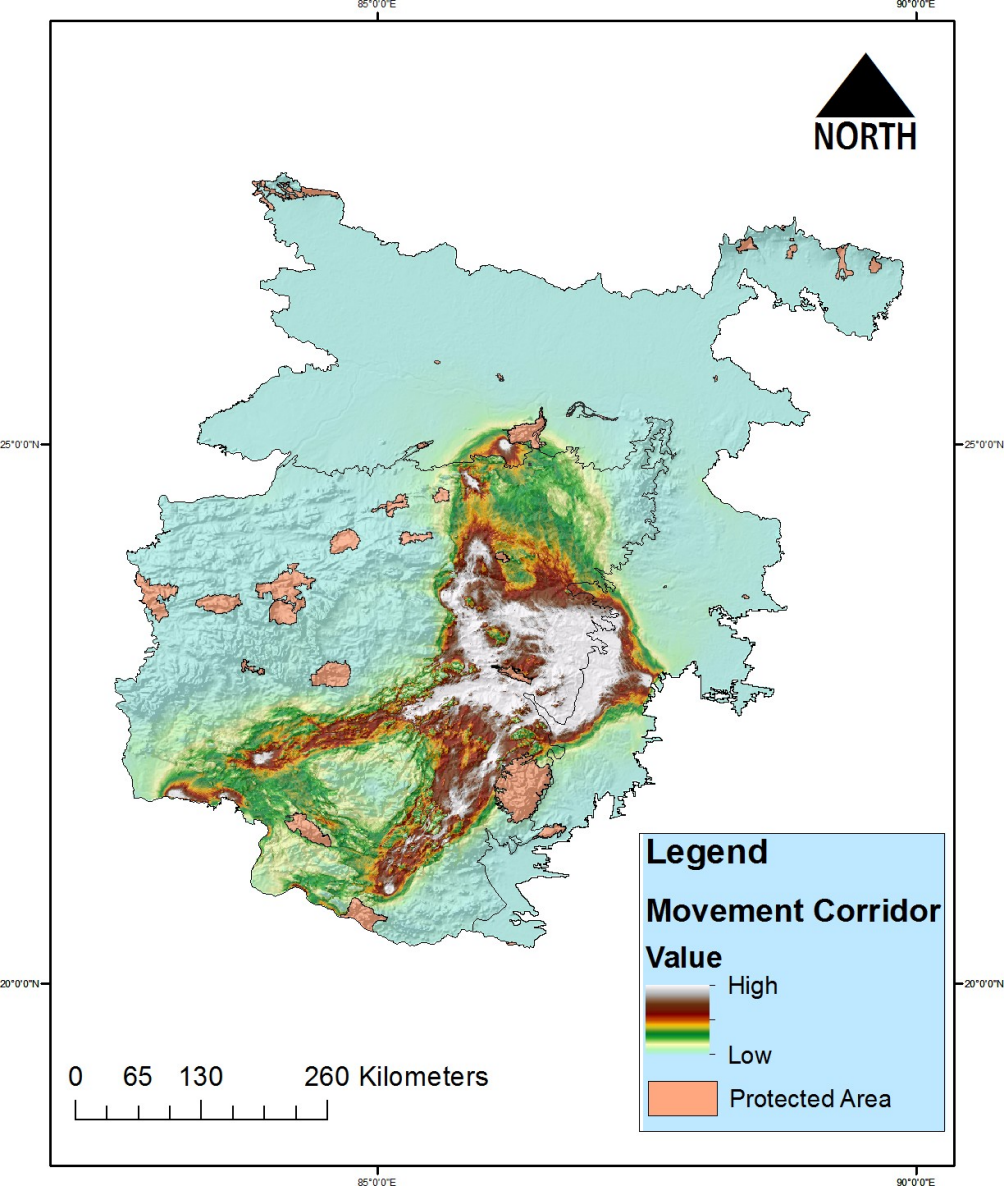
Map showing predicted movement corridors of Indian Grey Wolf in the study landscape.

The model indicates that much of the suitable habitats across the study landscape have biological connectivity. However, the cumulative current flow was highest in the zone which borders the two biotic provinces in the south-eastern side via districts of Purba Singhbhum and Paschim Singhbhum of Jharkhand and Bankura and West Midnapore districts of West Bengal. The present model also suggests that much of the connectivity exists in unprotected or territorial forest ranges in the landscape. However, two significant corridors have been identified which connects both biotic provinces, i.e., a corridor in the northern part of Chotta Nagpur Plateau via Bhimband and Koderma Range and other corridor is in the eastern face of the Chotta Nagpur plateau via districts of Bankura and West Mednipore (Fig 6). Among the PAs, a biological corridor between Simlipal NP-Satkosia WLS may also exist which is connecting the south Bengal with the Dalma range of Chotta Nagpur plateau. However, the model also indicates relatively weak connectivity may also exist between Koderma WLS, Khulasuni WLS and Debrigarh WLS.

## Discussion

The Indian grey wolf is one of the top carnivore species distributed in the open grasslands of peninsular India. Till date, much of the studies on the species have been conducted in its western and southern ranges, whereas, no reliable information is available in its eastern range except a short study by [8]. Furthermore, no attempt has been made to map its eastern range which is pro-vital for its conservation and management planning. The Indian grey wolf is threatened throughout its range due to its involvement in livestock depredation. The large tract of semi-arid eco-region is largely rainfed where the agriculture is mostly animal dependent and the economy of the local communities is based on animal husbandry. The increase in livestock depredation incidences by wolves is leading to the development of antagonistic behaviour among the locals towards the species which will be detrimental for its long-term survival [20, 58]. Hence, through the present model, we identified and mapped the habitats suitable for the species and much of the highly suitable habitats are falling in areas compost of dry scrub vegetation, open forest and agroforestry landscape [38]. The higher AUC value of 0.981 indicates that the selected variables in the present model were very good predictors of mapping suitable habitat of the species (S3 Fig). The negative association of the precipitation of the driest quarter and seasonality indicates that the suitable habitats for the species are located in areas with relatively drier conditions with the low amount of precipitation. The present results corroborate with the findings of the other studies highlighting that the wolf is a top carnivore reported to be distributed in areas with hot and semi-dry environmental conditions [3, 4, 5, 10].

The results indicate that out of the total area (14,476.61 Km^2^) under PA network, only 1,332 Km^2^ area was found to be suitable, suggesting that most of the suitable areas of the species were outside the PA areas of the landscape, which is one of the vital reasons for increasing human-wolf conflict. The wolf thrives well in Non-PA areas with relatively poor natural prey base [5, 11]. Moreover, previous it has been documented that the wolf population in Gujarat and Rajasthan states of India are surviving on livestock because of poor availability of wild prey species [5, 9, 59]. In the present scenario based on the interviewed villagers and experienced forest guard the population of wolves is on decrease due to retaliatory killings, illegal hunting, and habitat degradation.

The forest biodiversity conservation and management agencies in India have adopted several conservation majors, but most of them have been applied in PAs with focus on other large charismatic mammals such as tiger, leopard and elephants. However, the wolf which occupies an area which is mainly outside the PA network needs a differential management strategy. The earlier researchers have also suggested conservation, and management strategies for Indian Grey wolf which may be applicable throughout the most of its distribution ranges across India [4, 9, 10]. Its hardiness and great dispersal ability make this species to survive in agroforestry as well as in degraded habitats. It is a species which has evolved to thrive in dry and resources poor landscape hence demands strategies through which the natural composition of the landscapes can be mentioned without altering the structural configuration of the habitats. The activities such as habitat improvement through plantation or alternation in the landscape configuration may not be useful or may results in creating stress to the species [11].

Moreover, the prevailing concept of concentrated management in PAs includes Wildlife Sanctuaries, and National Park may not suffice the long-term conservation and management of the wolf population in India. The conservation strategies for wolf should not be restricted to some small patches of vast landscape, instead focus should be given on protecting the natural composit of its habitat which will promote the smooth functioning of the biological corridors and connectivity between the habitat patches. We suggest that the two identified corridors connecting Chotta Nagpur Plateau and Lower Gangetic plans are via Bhimband and Koderma Range and other from Bankura and West Mednipore will be vital for the long-term viability of the wolf populations may be prioritized for management interventions (Fig 6). The wolves are great dispersers and know to travel long distance for which they negotiate human disturbance. However, land use change and other anthropogenic disturbances can lead to negative impacts and also pose mortality risk to the species. At fine scale the habitat and landscape utilization of the species may get influence with habitat characters and anthropogenic disturbance [37, 60]. The non-PA forestry landscape management documents should have effective treatments focusing on wolves. In Indian forest management system, PAs are managed with focus on flagship faunal species whereas the non-PAs or the territorial forests are managed under the working plans. These working plans are focused on production forestry where treatment remedies are provided for enhancing the productivity of the forests. Hence, considering the fact that wolves live on both non-PAs and PAs there is a need to have differential management needs.

## Differential management needs of Indian Grey wolf (*Canis lupus pallipes)* in Chotta Nagpur Plateau and Lower Gangetic Plans

Although the two biotic provinces Chotta Nagpur Plateau and Lower Gangetic Plans represents two bio-geographic zones, i.e. Gangatic plans and Deccan Plateau but the border areas of these two provinces possess noteworthy commonality in terms of faunal species composition, topography, and climate as well as forest types. The present study has highlighted that the border area of the two provinces provides habitat which is supporting the remnant population of wolves in this landscape. The study could be also able to map the possible biological corridors through which the species may be using as movement corridors and utilizing the habitats.

In India the PAs and non-PA areas are managed with different aims and objectives, which are sometimes not complementary, and the species such as wolf which inhabit in the composit of both the types of areas in a landscape suffers. Moreover, while far-reaching the scopes, the implementation of the existing mechanism are exceedingly slow and not as productive as expected. Hence, urgent steps are needed to adopt landscape-level management planning for the long-term viability of wolf populations in the region. The working plans of the territorial forests in such areas should be developed with adequate conservation and management remedies for the species.

The Indian National Working Plan Code 2014 (NWPC 2014) for forest management is primarily based on the scientifically collected data relating to the growth, biodiversity, crop composition, forest biomass and their management strategies [61] but the efforts to link it with wildlife management objectives and practices have yet to come to desired levels. The NWPC 2014 should have a component which dealing with population management of flagship species such as wolf by retaining the original composition and the structure of the forested habitats through uneven and selection type forests. Since, earlier studies have established that wolf occupies areas with relatively open canopy forest patches and the management activity such as afforestation or changing the natural structure and composition of the forests may negatively impact the species [10]. Hence, we strongly propose changes in the National Working Plan Code 2014 (NWPC 2014) guidelines so that the wildlife management components could be given enough space in the document. Further, we also suggest the participation of local community in management planning should take centre stage so that the biological functionality of the identified wolf corridors in the landscape can be mentained for the long-term genetic viability of wolves. The capacity enhancement and mass awareness creation among the local communities will lead to minimize wolf-human conflict and also it will be helpful in developing compassionate attitude among the locals towards the species in the region.

Further, considering the fact that the species is patchy in distribution and non-availability of quality fine-scale data on habitat ecology of the species we suggest a long-term study covering the entire distribution range in the region. Moreover, we strongly recommend landscape genetic study on the species to understand the biological functionality of the corridors identified in the present study for the long-term conservation and management of the species.

## Supporting information

S1 Fig Forest cover map of CNP and LGP. (DOC)

S2 Fig Elevation map of CNP and LGP. (DOC)

S3 Fig The average training ROC for the replicate runs is 0.981, and the standard deviation is 0.007. (DOC)

S4 Fig Percentage contribution and permutation importance of selected variables. (DOC)

S5 Fig Response curves of the important variables for habitat suitability of gray wolf. Curves shows how logistic prediction of the model changes with the selected variables. Keeping all other variables at their average sample value.
(DOC)

S6 Fig 100% staked bar chart of open-ended semi-structured questionnaire survey. Representing the relative percentage of multiple questionnaire survey series along with the no. of respective respondent. Black bar = indicates the respondents aggress with the statement, Grey bar = indicates the respondents who doesn’t aggress with the statement.
(DOC)

S1 Table Accuracy assessment table for forest cover classification of CNP and LGP. (DOC)

S2 Table Zonal statistics table for protected areas in CNP and LGP. (DOC)

S3 Table List of all 23 variables for habitat suitability modelling for Indian Grey Wolf (*Canis lupus pallipes*) in the study landscapes (Cotta Nagpur Plateau and Lower Gangetic Plain). (DOC)
