# Supplementary figures and images for: Identifying suitable habitat and corridors for Indian Grey Wolf (Canis lupus pallipes) in Chotta Nagpur Plateau and Lower Gangetic Planes: A species with differential management needs
Source: PLoS One. 2019 Apr 10;14(4):e0215019. doi: 10.1371/journal.pone.0215019 (PMC6457547; doi:10.1371/journal.pone.0215019)

**S1 Fig: Forest cover map of CNP and LGP.**


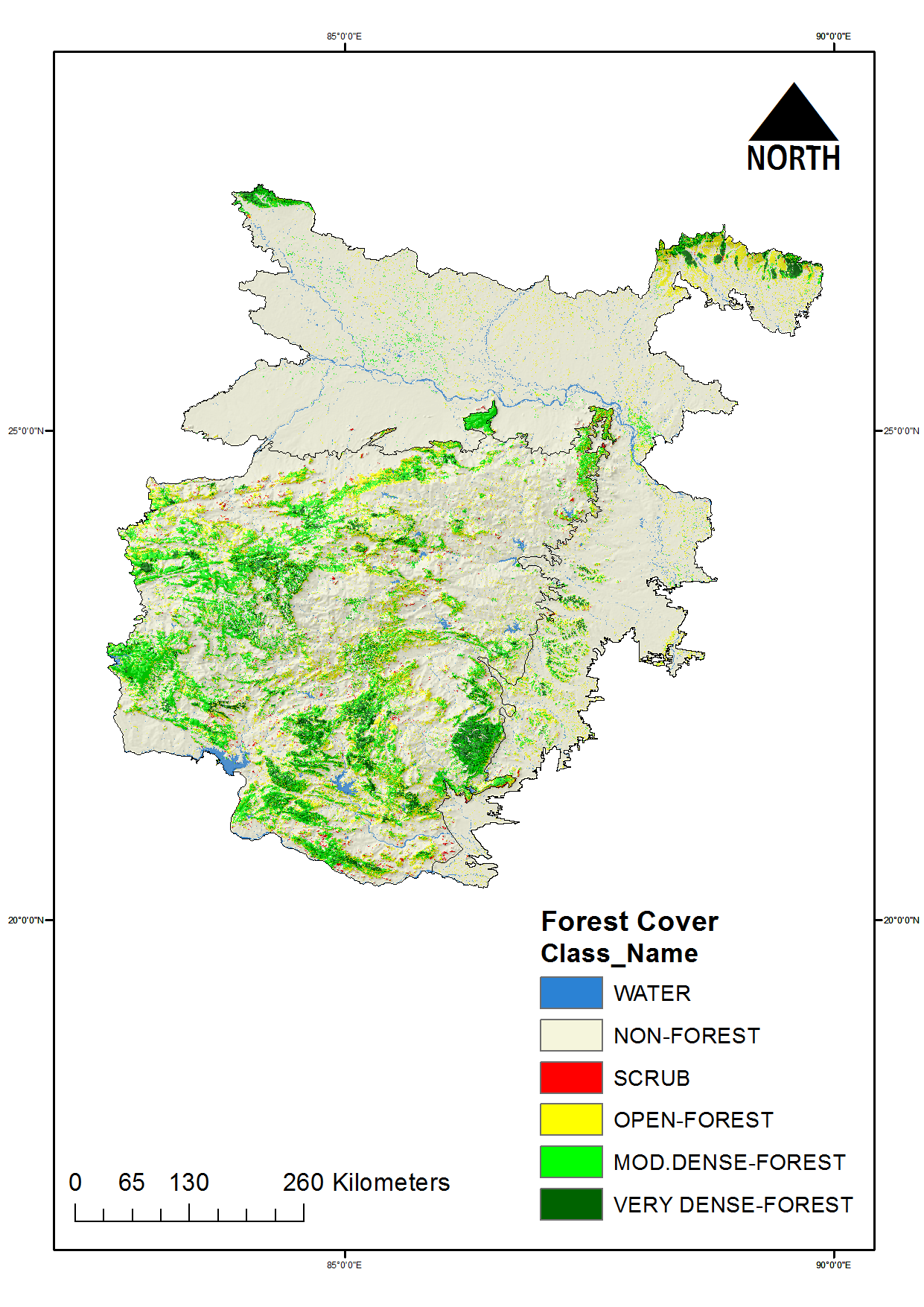

Supplement: S1 Fig — (DOC) [file pone.0215019.s001.doc]

**S2 Fig. Elevation map of CNP and LGP.** Scales showing the altitude ranging from low (green) to high (red).

**
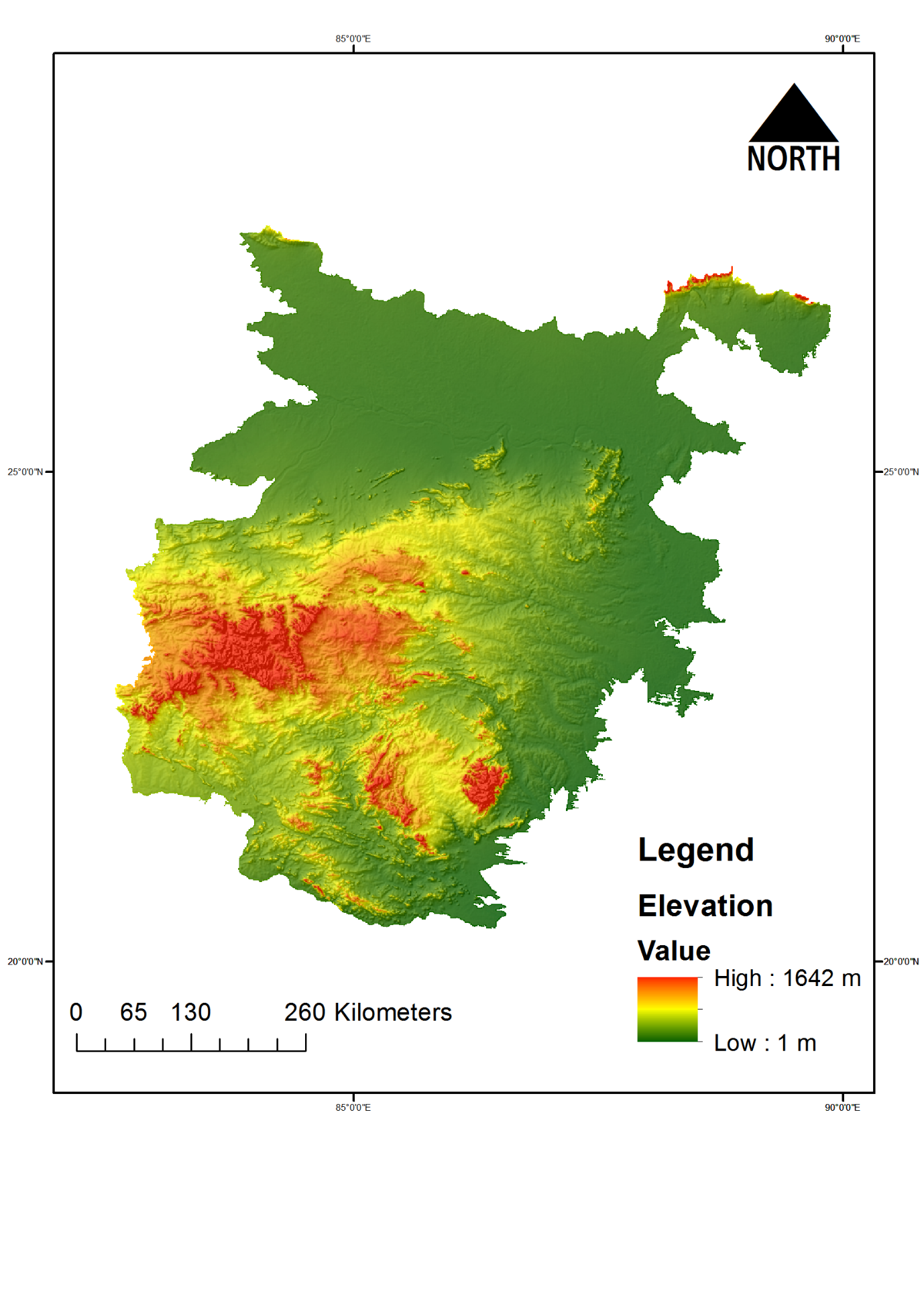
**

Supplement: S2 Fig — (DOC) [file pone.0215019.s002.doc]

**S3 Fig. The average training ROC for the replicate runs is 0.981, and the standard deviation is 0.007.**


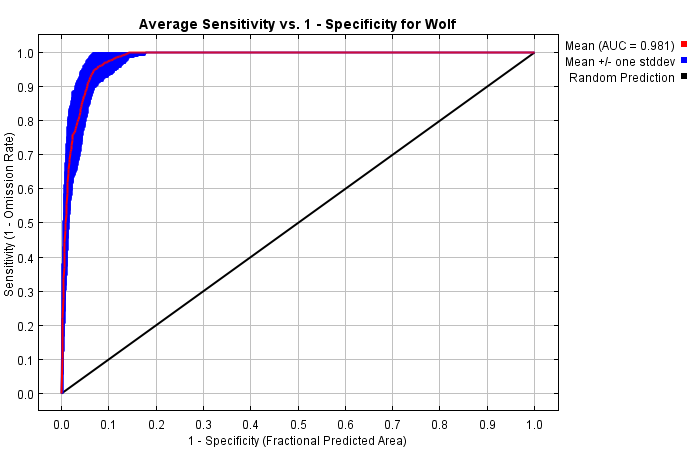

Supplement: S3 Fig — (DOC) [file pone.0215019.s003.doc]
