# Supplementary material for: Identifying suitable habitat and corridors for Indian Grey Wolf (Canis lupus pallipes) in Chotta Nagpur Plateau and Lower Gangetic Planes: A species with differential management needs
Source: PLoS One. 2019 Apr 10;14(4):e0215019. doi: 10.1371/journal.pone.0215019 (PMC6457547; doi:10.1371/journal.pone.0215019)
Supplement: S4 Fig — (DOC) [file pone.0215019.s004.doc]

**S4 Fig. Percentage contribution and permutation importance of selected variables.**
