# Supplementary material for: Identifying suitable habitat and corridors for Indian Grey Wolf (Canis lupus pallipes) in Chotta Nagpur Plateau and Lower Gangetic Planes: A species with differential management needs
Source: PLoS One. 2019 Apr 10;14(4):e0215019. doi: 10.1371/journal.pone.0215019 (PMC6457547; doi:10.1371/journal.pone.0215019)
Supplement: S5 Fig — Curves shows how logistic prediction of the model changes with the selected variables. Keeping all other variables at their average sample value. (DOC) [file pone.0215019.s005.doc]

**S5 Fig. Response curves of the important variables for habitat suitability of gray wolf. Curves shows how logistic prediction of the model changes with the selected variables. Keeping all other variables at their average sample value.**

| 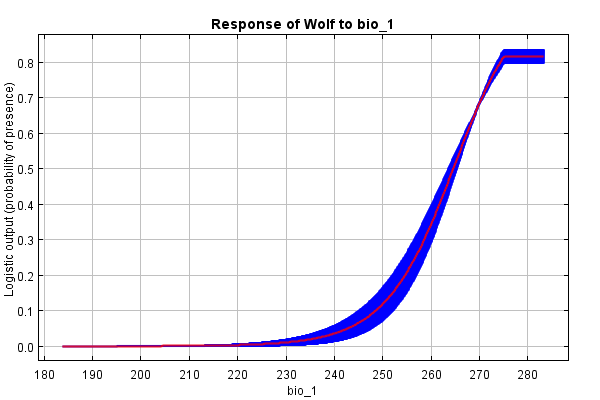  A | 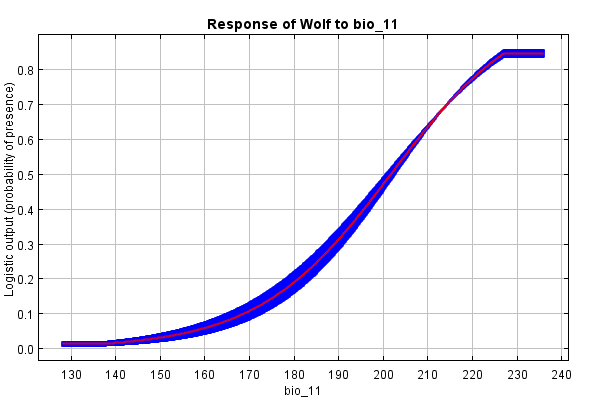  B |
| --- | --- |
| 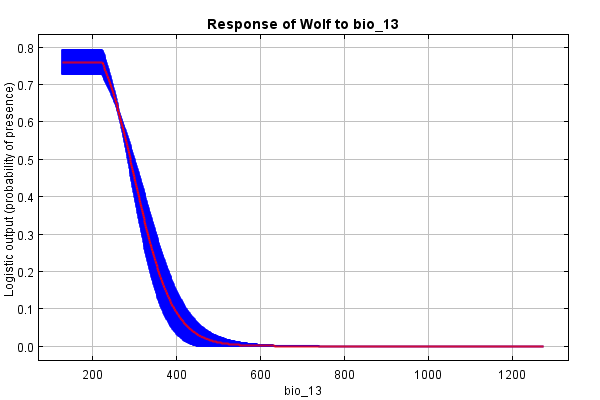  G  C | 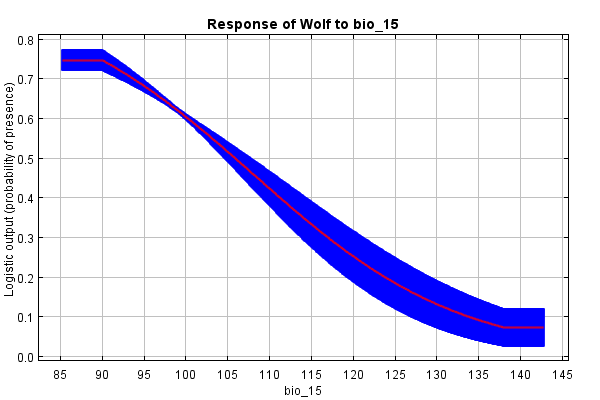  D |
| 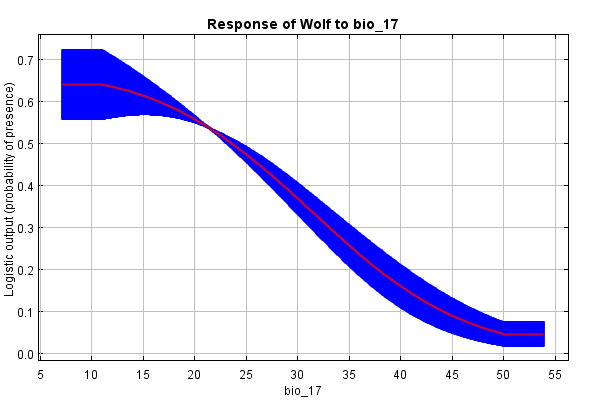  E | 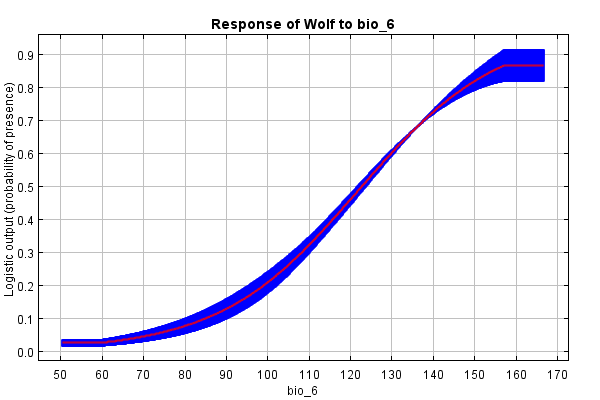  F |
| 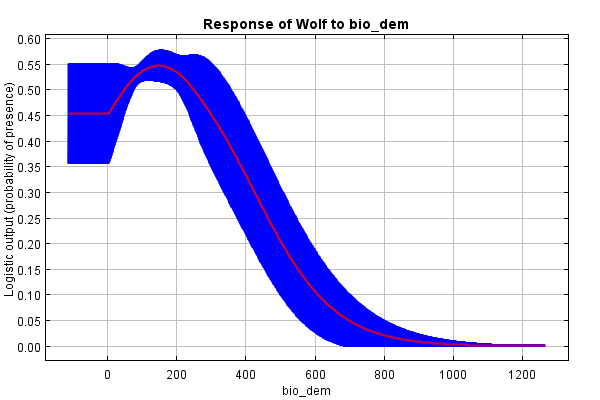 |  |
