# Supplementary material for: Identifying suitable habitat and corridors for Indian Grey Wolf (Canis lupus pallipes) in Chotta Nagpur Plateau and Lower Gangetic Planes: A species with differential management needs
Source: PLoS One. 2019 Apr 10;14(4):e0215019. doi: 10.1371/journal.pone.0215019 (PMC6457547; doi:10.1371/journal.pone.0215019)

**S6 Fig. 100% staked bar chart of open-ended semi-structured questionnaire survey.** Representing the relative percentage of multiple questionnaire survey series along with the no. of respective respondent. Black bar = indicates the respondents aggress with the statement, Grey bar = indicates the respondents who doesn’t aggress with the statement.


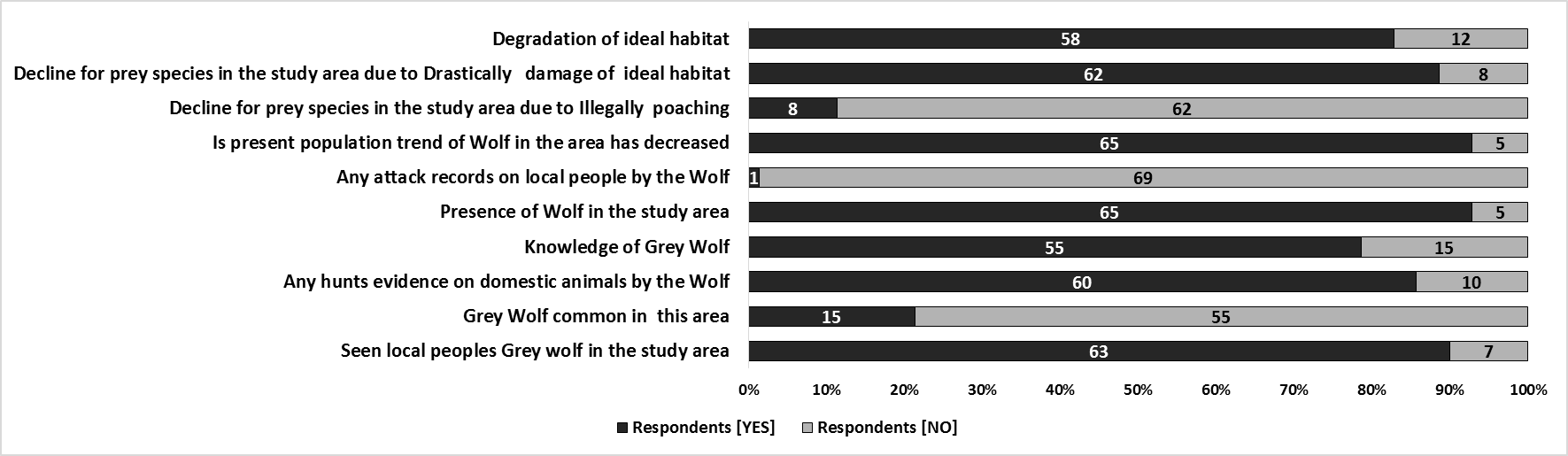

Supplement: S6 Fig — Representing the relative percentage of multiple questionnaire survey series along with the no. of respective respondent. Black bar = indicates the respondents aggress with the statement, Grey bar = indicates the respondents who doesn’t aggress with the statement. (DOC) [file pone.0215019.s006.doc]
