# Supplementary material for: Identifying suitable habitat and corridors for Indian Grey Wolf (Canis lupus pallipes) in Chotta Nagpur Plateau and Lower Gangetic Planes: A species with differential management needs
Source: PLoS One. 2019 Apr 10;14(4):e0215019. doi: 10.1371/journal.pone.0215019 (PMC6457547; doi:10.1371/journal.pone.0215019)
Supplement: S1 Table — (DOC) [file pone.0215019.s007.doc]

**S1** Table. Accuracy assessment table for forest cover classification of CNP and LGP.

| **Forest cover classification of CNP and LGP** | | | | | | | | | |
| --- | --- | --- | --- | --- | --- | --- | --- | --- | --- |
| **Contingency Matrix** | | | | | | | | | |
|  | | **Observed Vegetation Classes** | | | | | | **Grand Total:** | **User's Accuracy:** |
|  |  | **WATER** | **NON-FOREST** | **SCRUB** | **OPEN-FOREST** | **MOD. DENSE FOREST** | **VERY DENSE FOREST** |  |  |
| **Mapped Vegetation Classes:** | **WATER** | **55** | **3** | **0** |  | **0** | **0** | **58** | **94.82758621** |
|  | **NON-FOREST** | **3** | **57** | **0** | **3** | **0** | **0** | **63** | **90.47619048** |
|  | **SCRUB** | **2** | **0** | **51** | **9** | **3** | **0** | **65** | **78.46153846** |
|  | **OPEN-FOREST** | **0** | **0** | **6** | **48** | **0** | **2** | **56** | **85.71428571** |
|  | **MOD. DENSE FOREST** | **0** | **0** | **3** | **0** | **53** | **3** | **59** | **89.83050847** |
|  | **VERY DENSE FOREST** | **0** | **0** |  | **0** | **4** | **55** | **59** | **93.22033898** |
| **Grand Total:** | | **60** | **60** | **60** | **60** | **60** | **60** | **360** |  |
| **Producer's Accuracy:** | | **91.666667** | **95** | **85** | **80** | **88.333333** | **91.666667** |  |  |
| **Samples: 360 Overall Accuracy: 88.61% Kappa Statistic: 86.30%** | | | | | | | | | |
| | **Kappa Coefficient** | | --- | | Number of observed agreements: 319 ( 88.61% of the observations) | | Number of agreements expected by chance: 60.0 ( 16.67% of the observations) | | Kappa= 0.863 | | SE of kappa = 0.020 | | 95% confidence interval: From 0.824 to 0.903 | | The strength of agreement is considered to be 'very good'. | | The calculation of weighted kappa, below, assumes the categories are ordered and accounts for  how far apart the two raters are. This calculation uses linear weights. | | Weighted Kappa= 0.923 | | Assessed this way, the strength of agreement is considered to be 'very good'. | | | | | | | | | | |
