# Supplementary material for: Identifying suitable habitat and corridors for Indian Grey Wolf (Canis lupus pallipes) in Chotta Nagpur Plateau and Lower Gangetic Planes: A species with differential management needs
Source: PLoS One. 2019 Apr 10;14(4):e0215019. doi: 10.1371/journal.pone.0215019 (PMC6457547; doi:10.1371/journal.pone.0215019)
Supplement: S2 Table — (DOC) [file pone.0215019.s008.doc]

**S2 Table. Zonal statistics table** for protected areas in CNP and LGP

| **OBJECTID** | **PANAME** | **COUNT** | **AREA** | **MIN** | **MAX** | **RANGE** | **MEAN** | **STD** | **SUM** |
| --- | --- | --- | --- | --- | --- | --- | --- | --- | --- |
| **1** | Buxa NP | 156 | 0.0108333 | 1.97034E-09 | 3.31115E-05 | 3.31095E-05 | 4.32029E-06 | 7.02567E-06 | 0.000673965 |
| **2** | Jaldapara WLS | 304 | 0.0211111 | 1.46883E-08 | 0.000252181 | 0.000252166 | 2.95083E-05 | 4.26755E-05 | 0.00897054 |
| **3** | Neora Valley NP | 18 | 0.00125 | 2.23127E-08 | 1.0209E-07 | 7.97773E-08 | 3.79408E-08 | 1.7501E-08 | 6.82934E-07 |
| **4** | Gorumara NP | 104 | 0.00722222 | 4.38476E-06 | 4.55036E-05 | 4.11188E-05 | 2.17968E-05 | 1.20875E-05 | 0.00226686 |
| **5** | Mahananda WLS | 202 | 0.0140278 | 5.60932E-08 | 0.000137657 | 0.000137601 | 2.83646E-05 | 2.77826E-05 | 0.00572965 |
| **6** | Chapramari WLS | 15 | 0.00104167 | 1.88823E-06 | 7.62278E-06 | 5.73455E-06 | 3.09822E-06 | 1.57376E-06 | 4.64733E-05 |
| **7** | Ballavpur WLS | 4 | 0.000277778 | 0.0353463 | 0.0666587 | 0.0313124 | 0.047551 | 0.0115977 | 0.190204 |
| **8** | Raiganj WLS | 13 | 0.000902778 | 0.00226236 | 0.0049722 | 0.00270984 | 0.0034922 | 0.000708339 | 0.0453986 |
| **9** | Bethuadahari WLS | 23 | 0.00159722 | 0.0141528 | 0.0267297 | 0.0125769 | 0.0226641 | 0.00411307 | 0.521274 |
| **10** | Sohagibarwa WLS | 14 | 0.000972222 | 3.6395E-06 | 1.79698E-05 | 1.43303E-05 | 7.15481E-06 | 4.54113E-06 | 0.000100167 |
| **11** | Badrama | 578 | 0.0401389 | 0.000345558 | 0.00720972 | 0.00686416 | 0.00193634 | 0.000966614 | 1.1192 |
| **12** | Khalasuni WLS | 263 | 0.0182639 | 0.000470844 | 0.0268878 | 0.026417 | 0.00596574 | 0.00615961 | 1.56899 |
| **13** | Kuldiha WLS | 284 | 0.0197222 | 1.73818E-06 | 0.000202625 | 0.000200887 | 3.52711E-05 | 2.77048E-05 | 0.010017 |
| **14** | Hadgarh WLS | 20 | 0.00138889 | 0.00119447 | 0.00357094 | 0.00237647 | 0.00231239 | 0.000648145 | 0.0462478 |
| **15** | Debrigarh WLS | 3 | 0.000208333 | 0.0326105 | 0.0764636 | 0.0438531 | 0.0500799 | 0.0189802 | 0.15024 |
| **16** | Satkosia WLS | 772 | 0.0536111 | 0.000253011 | 0.245513 | 0.24526 | 0.0514988 | 0.0450592 | 39.7571 |
| **17** | Simlipal NP | 1152 | 0.08 | 0.000011724 | 0.0420385 | 0.0420268 | 0.00180073 | 0.00472916 | 2.07444 |
| **18** | Simlipal WLS | 1806 | 0.125417 | 4.08101E-05 | 0.132173 | 0.132132 | 0.00801573 | 0.0140047 | 14.4764 |
| **19** | Lawalong WLS | 625 | 0.0434028 | 2.99241E-05 | 0.000255808 | 0.000225884 | 0.000095849 | 4.24945E-05 | 0.0599056 |
| **20** | Hazaribagh WLS | 291 | 0.0202083 | 6.94625E-05 | 0.0067538 | 0.00668434 | 0.000759295 | 0.000829723 | 0.220955 |
| **21** | Palkot WLS | 954 | 0.06625 | 1.15092E-06 | 0.00141037 | 0.00140922 | 0.000103235 | 0.000201075 | 0.098486 |
| **22** | Mahauaduar WLS | 623 | 0.0432639 | 7.15007E-06 | 0.000298112 | 0.000290962 | 6.03432E-05 | 4.73403E-05 | 0.0375938 |
| **23** | Koderma WLS | 198 | 0.01375 | 0.0092356 | 0.0925086 | 0.083273 | 0.0247449 | 0.0154785 | 4.89948 |
| **24** | Gautam Budha á á | 140 | 0.00972222 | 0.00160186 | 0.0311144 | 0.0295125 | 0.0115897 | 0.0066119 | 1.62256 |
| **25** | Dalma WLS | 304 | 0.0211111 | 0.000803401 | 0.575383 | 0.57458 | 0.166906 | 0.117601 | 50.7393 |
| **26** | Palamau WLS | 1107 | 0.076875 | 5.35782E-06 | 0.000441313 | 0.000435955 | 5.29116E-05 | 5.69883E-05 | 0.0585731 |
| **27** | Parasnath WLS | 83 | 0.00576389 | 4.20622E-05 | 0.121817 | 0.121775 | 0.0307513 | 0.0268085 | 2.55236 |
| **28** | Topchanchi WLS | 26 | 0.00180556 | 0.0333389 | 0.0975771 | 0.0642382 | 0.063728 | 0.0180329 | 1.65693 |
| **29** | Betla NP | 312 | 0.0216667 | 5.67325E-06 | 0.000272545 | 0.000266872 | 2.94744E-05 | 3.49666E-05 | 0.009196 |
| **30** | Semarsot WLS | 759 | 0.0527083 | 2.36087E-06 | 8.04773E-05 | 7.81164E-05 | 2.39922E-05 | 1.20819E-05 | 0.0182101 |
| **31** | Tamorpingla WLS | 1069 | 0.0742361 | 3.1244E-06 | 0.000107076 | 0.000103952 | 2.56543E-05 | 1.68331E-05 | 0.0274245 |
| **32** | Badalkhol WLS | 146 | 0.0101389 | 0.000093889 | 0.00221062 | 0.00211673 | 0.000453676 | 0.000252544 | 0.0662366 |
| **33** | Vikramshila Ganges WLS | 113 | 0.00784722 | 0.000741299 | 0.0399042 | 0.0391629 | 0.0170661 | 0.0110764 | 1.92847 |
| **34** | Rajgir pant WLS | 64 | 0.00444445 | 0.00255469 | 0.0835133 | 0.0809586 | 0.0302208 | 0.0192313 | 1.93413 |
| **35** | Gautam buddha WLS | 219 | 0.0152083 | 0.000391663 | 0.027124 | 0.0267323 | 0.00924353 | 0.00671181 | 2.02433 |
| **36** | Kanwarjheel WLS | 33 | 0.00229167 | 0.00282185 | 0.00669551 | 0.00387366 | 0.0047679 | 0.00105251 | 0.157341 |
| **37** | Bhimbandh WLS | 884 | 0.0613889 | 0.00285286 | 0.389211 | 0.386358 | 0.056628 | 0.0571225 | 50.0591 |
| **38** | Barela S.A.Z.S. WLS | 16 | 0.00111111 | 0.0135825 | 0.0257342 | 0.0121517 | 0.0193222 | 0.00317324 | 0.309155 |
| **39** | Kaimur WLS | 11 | 0.000763889 | 0.015932 | 0.0418686 | 0.0259366 | 0.0254426 | 0.00661698 | 0.279868 |
| **40** | Valmiki WLS | 715 | 0.0496528 | 6.02841E-08 | 0.000179818 | 0.000179758 | 7.28215E-06 | 1.81405E-05 | 0.00520674 |
| **41** | Valmiki NP | 3 | 0.000208333 | 2.67133E-06 | 4.3501E-06 | 1.67877E-06 | 3.71999E-06 | 7.46528E-07 | 0.00001116 |
| **42** | Nagi Dam WLS | 4 | 0.000277778 | 0.0938003 | 0.140711 | 0.0469107 | 0.119922 | 0.02049 | 0.479686 |
