# Supplementary material for: Identifying suitable habitat and corridors for Indian Grey Wolf (Canis lupus pallipes) in Chotta Nagpur Plateau and Lower Gangetic Planes: A species with differential management needs
Source: PLoS One. 2019 Apr 10;14(4):e0215019. doi: 10.1371/journal.pone.0215019 (PMC6457547; doi:10.1371/journal.pone.0215019)
Supplement: S3 Table — (DOC) [file pone.0215019.s009.doc]

**S3 Table. List of all 23 variables for habitat suitability modelling for Indian Grey Wolf (*Canis lupus pallipes*) in the study landscape (Cotta Nagpur Plateau and Lower Gangetic Plain)**

| **Variables** | **Code** | **Type** |
| --- | --- | --- |
| Bio 1 = Annual Mean Temperature | Bio_1 | Continuous |
| Bio 2 = Mean Diurnal Range (Mean of monthly (max temp - min temp)) | Bio_2 | Continuous |
| Bio 3 = Isothermality (BIO2/BIO7) | Bio_3 | Continuous |
| Bio 4 = Temperature Seasonality ( Standard Deviation ) | Bio_4 | Continuous |
| Bio 5 = Max Temperature of Warmest Month | Bio_5 | Continuous |
| Bio 6 = Minimum Temperature of Coldest Month | Bio_6 | Continuous |
| Bio 7 = Temperature Annual Range (BIO5-BIO6) | Bio_7 | Continuous |
| Bio 8 = Mean Temperature of Wettest Quarter | Bio_8 | Continuous |
| Bio 9 = Mean Temperature of Driest Quarter | Bio_9 | Continuous |
| Bio 10 = Mean Temperature of Warmest Quarter | Bio_10 | Continuous |
| Bio 11 = Mean Temperature of Coldest Quarter | Bio_11 | Continuous |
| Bio 12 = Annual Precipitation | Bio_12 | Continuous |
| Bio 13 = Precipitation of Wettest Month | Bio_13 | Continuous |
| Bio 14 = Precipitation of Driest Month | Bio_14 | Continuous |
| Bio 15 = Precipitation of Seasonality (Coefficient of Variation) | Bio_15 | Continuous |
| Bio 16 = Precipitation of Wettest Quarter | Bio_16 | Continuous |
| Bio 17 = Precipitation of Driest Quarter | Bio_17 | Continuous |
| Bio 18 = Precipitation of Warmest Quarter | Bio_18 | Continuous |
| Bio 19 = Precipitation of Coldest Quarter | Bio_19 | Continuous |
| DEM = Digital elevation data (m) from Advanced Spaceborne Thermal Emission & Reflection Radiometer (ASTER) | Bio_DEM | Continuous |
| River = Euclidian distance (m) from River | Bio_River | Continuous |
| Road = Euclidian distance (m) from River | Bio_Road | Continuous |
| Forest Cover   1. Dense forest 2. Moderate dense forest 3. Open forest 4. Scrubs 5. Non-forest 6. Water | Bio_veg | Categorical |
